# Nonfunctional double parathyroid carcinoma with incidental thyroid micropapillary carcinoma: a rare case

**DOI:** 10.11604/pamj.2017.27.241.11503

**Published:** 2017-08-02

**Authors:** Kursat Dikmen, Hasan Bostanci, Huseyin Gobut, Alp Yildiz, Onur Ertunc, Ali Celik, Murat Akin, Ferit Taneri

**Affiliations:** 1 Department of General Surgery, Gazi University Medical Faculty, Ankara, Turkey; 2 Department of Pathology, Gazi University Medical Faculty, Ankara, Turkey; 3 Department of Thoracic Surgery, Gazi University Medical Faculty, Ankara, Turkey

**Keywords:** Non-functional parathyroid carcinoma, double parathyroid carcinoma, thyroid papillary carcinoma

## Abstract

Parathyroid carcinomas are rare endocrine tumors which comprise 0.3-5.6% of all causes of hyperparathyroidism. 90% of them are hormonally active, while 10% of them may be non-functional. They mostly occur in a single parathyroid gland. Concurrent involvement of both parathyroid glands is quite rare. A 57-year-old male patient was admitted to emergency department with the complaint of dyspnea. Thorax tomography revealed a retrosternal mass. The mass was thoracoscopically excised by thoracic surgeons. Histopathological examination result of the mass was reported as parathyroid carcinoma. Parathyroid scintigraphy performed and focal activity increase in the lower pole of the left lobe. Parathyroid hormone level was 118 pg/ml and calcium level was measured as 11.4 mg/dl. The patient with these findings was operated and pathological examination of excised left lower parathyroid tissue was reported as carcinoma. In addition, micropapillary carcinoma was detected in left thyroid lobectomy specimen.Our case was also unusual in that double parathyroid carcinoma, which is a rare condition, was hormonally inactive. We aimed to present our case in the light of the literature due to its rare occurrence.

## Introduction

The most common causes of primary hyperparathyroidism are adenoma and hyperplasia, while carcinoma is rarely seen with a prevalence of 0.3-5.6% [1, 2]. Parathyroid carcinomas are generally hormonally active, about 10% of them are non-functional [3, 4]. In hormonally active patients, increased levels of parathyroid hormone (PTH) (8-10 times the normal value), calcium (>14 mg/dl) and alkaline phosphatase are specific. However, there is a group of patients with hormonally inactive disease in which calcium and PTH levels are normal or minimally increased [5–7]. Only 1 case has been reported in the literature, which concurrently had this rare endocrine malignancy, and histologically proven double carcinoma [8]. Furthermore, co-occurrence of parathyroid and thyroid pathologies is also a rare condition and it is mostly detected incidentally after surgery [9–11]. There is no study in the literature reporting co-occurrence of non-functioning parathyroid carcinoma involving both sides and thyroid micropapillary carcinoma. For this reason, we aimed to present our case in the light of the literature.

## Patient and observation

**Figure 1:**
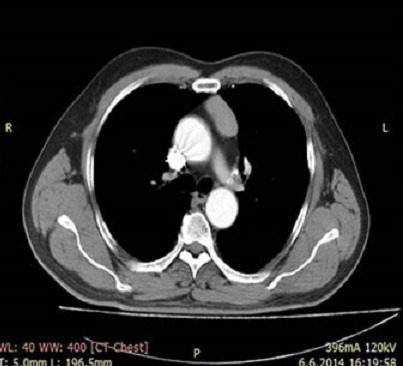
Preoperative tomography image of the mass lateral to sternum at the left

57-year old male patient was admitted to emergency department with dyspnea and tachypnea. Thorax computed tomography (CT) examination was performed with the initial diagnosis of pulmonary embolism, which revealeda 3x2 cm sized mass located lateral to sternum in the left side of anterior mediastinum (Figure 1). The mediastinal mass was thoracoscopically removed by en bloc excision. Since histopathological examination revealed parathyroid carcinoma, general surgery consultation was made.

On physical examination, there was no palpable mass in both thyroid lobes and no cervical lymph nodes. Review of organ systems revealed nothing pathological. He had no history of previous surgical operation and regular drug treatment. Laboratory parameters were as follows; Calcium: 11.4 mg/dl, phosphorus: 1.79 mg/dl, parathyroid hormone: 118.7 pg/ml, calcitonin: < 2 pg/ml and 25 hydroxy vitamin D: 21.84 ug/l. Neck ultrasonography (USG) detected a 25x11 mm sized lesion which was adjacent to left thyroid lobe inferoposteriorly, extending caudally and had cystic areas. This lesion had moderate level vascularity on Doppler Ultrasonography. The remaining of thyroid tissue was normal and no pathological lymph node was detected. Neck and thorax diffusion MR examinations demonstrated a 21x11 mm sized cystic lesion in parathyroid region, which was located posterior to lower part of left thyroid lobe and had clear margins. There was no suspicious appearance in the region of previous operation. Technetium-99m methoxy-isobutylisonitrile(Tc-99m MIBI) parathyroid scintigraphic examination was performed which showed early and late phase focal activity increase adjacent to lower pole of left lobe. On whole body bone densitometry, AP spine bone mineral density values (T score) and age matched (Z score) value revealed osteopenia, while right proximal femoral neck bone mineral density values (T score) showed osteopenia and age matched value (Z score) was normal. Laboratory parameters on the day before the operation were as follows; Calcium: 11.1 mg/dl, phosphorus: 2.39 mg/dl, PTH: 184 pg/ml, thyroid function tests and thyroglobulin levels: normal.

**Figure 2:**
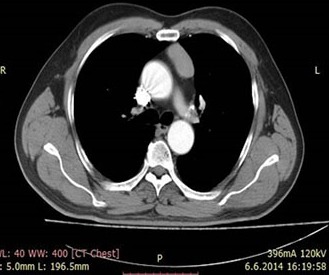
A) 25x10 magnification H&E and parathyroid pathology section; stained border surrounding connective tissue and partly capsule (arrow); B) 4x10 magnification H&E tumoral area in surrounding connective tissue (asterisk)

**Figure 3:**
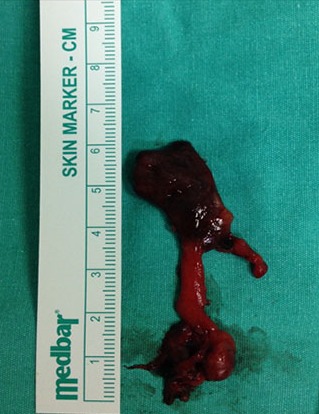
Intraoperative macroscopic appearance of left upper and lower parathyroid glands

Left inferior parathyroid gland peroperatively appeared bigger than normal and connected to superior parathyroid gland by a band (Figure 2). Both parathyroid tissues were excised and it was confirmed by frozen that the excised tissues were parathyroid glands. Intact PTH values measured 15 and 30 minutes after parathyroid excision were 78 and 52 pg/ml, respectively. Since intact PTH (iPTH) levels decreased by 70% of pre-operative values and the appearance of other parathyroid glands were not pathological, the operation was terminated after left thyroid lobectomy, isthmectomy+unilateral central lymph node dissection were performed (Figure 3). Serum calcium and PTH values measured 24 hours after the operation were 9.2 mg/dl and 63 pg/ml, respectively.

### Histopathological evaluation

**Figure 4:**
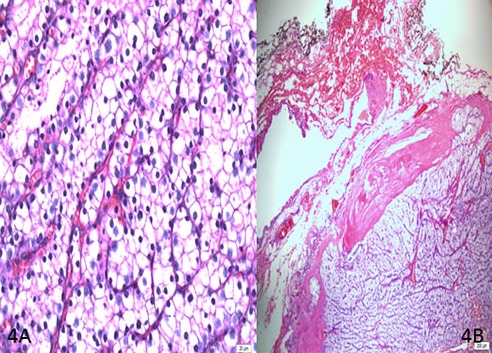
A) 40x10 magnification H&E, note clear cytoplasm, marked cell membrane, eccentric cell nucleus, patchy nuclear hypertrophy (arrow); cells are arranged as trabeculae and in clusters; B) 4x10 magnification H&E, arrows show peripheral thick capsule which was invaded by tumoral focuses that pass over the capsule to infiltrate surrounding connective tissue. Black capsule marked with asterisk is external surface surgical margin

**Figure 5:**
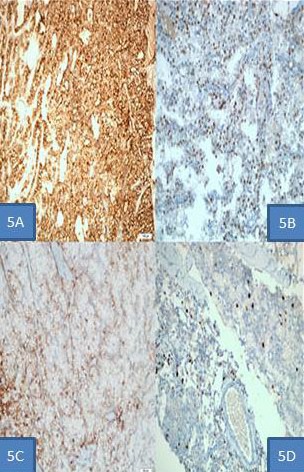
A) 10x10 streptavidin peroxidase staining, PTH immunohistochemical staining, diffuse strong cytoplasmic and membranous staining; B) 20x10 streptavidin peroxidase staining, cyclin D1 immunohistochemical staining, diffuse strong nuclear staining; C) 20x10 streptavidin peroxidase staining, Galectin-3 immunohistochemical staining, patchy strong cytoplasmic and nuclear staining; D) 20x10 streptavidin peroxidase staining, ki-67 immunohistochemical staining, note 2% nuclear staining

90% of the tumors had fibrous bands and trabecular structures seen in microscopic examination. 67% of them had capsular invasion, 12% showed vascular invasion, while mitosis and fusiform tumoral cells were present in 81% (Figure 2 and Figure 4). Extra-parathyroidal lesion was immunohistochemically excluded by means of PTH, TTF-1, PAX-8, HMB45, Melan-A, CD117, desmin and vimentin. We detected a strong staining by Galectin-3 and Cyclin-D1, which were used to differentiate parathyroid adenoma and carcinoma. 2% increase in proliferation was observed by Ki-67 (Figure 5).

## Discussion

The most common causes of primary hyperparathyroidism are adenoma and hyperplasia, while carcinoma is rarely seen with a prevalence of 0.3-5.6% [1, 2]. Parathyroid carcinoma is typically seen between the ages of 45 and 60 years with equal frequencies in both genders [7].

Although its etiopathology is not clear, increase in HRPT-2 tumor suppressor gene has been thought to play role in the etiology [12]. In addition, hyperparathyroidism may be seen sporadically or as a part of a genetic syndrome such as hyperparathyroidism jaw-tumor syndrome (HPT-JT), multiple endocrine neoplasia type-1 (MEN-1), MEN-2A and isolated familial hyperparathyroidism [7]. Although there are no established risk factors other than these genetic syndromes, it is more common in the patients with parathyroid gland hyperplasia related to chronic renal failure and radiation exposure to neck region [7]. In our case, parathyroid carcinoma was not a part of a genetic syndrome and the patient had none of the known risk factors for carcinoma.

Most of the parathyroid carcinomas are hormonally active, while 10% of them are non-functional [3, 4]. Increased levels of parathyroid hormone (8-10 times the normal value), calcium (>14 mg/dl) and alkaline phosphatase are usually detected in the patients with functional disease [5–7]. Clinical picture may include polyuria, renal colic, nephrolithiasis, bone pain, osteopenia, pathologic fractures, nausea, abdominal pain, peptic ulcer, pancreatitis, fatigue and depression [6, 7, 13]. In the patients with non-functional disease, hoarseness secondary to recurrent laryngeal nerve invasion, palpable neck mass, symptoms and signs of distant metastasis may be present in the absence of these symptoms [3, 4]. Since our case did not have classical clinical and laboratory findings of parathyroid carcinoma, he was accepted to have non-functional disease.

There is no specific imaging method that is used alone for parathyroid diseases. Technetium-99m Sestamibi scan and neck USG examinations are generally used [14]. In addition, imaging modalities such as neck magnetic resonance (MR) and CT are commonly used methods in detecting regional lymph nodes, invasion to surrounding tissues or distant organ metastasis [14]. In a study, sensitivities of sestamibi scan, MR, CT and USG examinations were reported to be 79%, 93%, 69% and 83%, respectively [14]. In our case, 20 mCiTc-99m MIBI parathyroid scintigraphic examination revealed focal activity increase in the lower pole of left thyroid lobe. A 20x10 mm sized lesion which was located in parathyroid region and had moderate level of vascularity was detected on neck and thyroid USG. MR examination performed for staging demonstrated a 21x11 mm sized cystic mass which was located in the left parathyroid region and showed weak peripheral contrast enhancement. Although the lesion was in a close relationship with cervical part of the esophagus, any evidence of infiltration was not observed.

Surgical treatment is the only curative modality for long term survival. In addition to the excision of pathological gland, ipsilateral thyroidectomy is also recommended [12, 13].In parathyroid carcinomas, the rates of invasion were 89% for ipsilateral thyroid gland, 71% for strap muscles, 26% for ipsilateral recurrent laryngeal nerve, 17% for esophagus and 17% for trachea [15]. Cervical lymph node involvement may be seen in about 15-30% of the cases [14, 15]. In case of pre-operative or intra-operative detection of lymph nodes, ipsilateral lymph node dissection is recommended after verification of metastasis in these lymph nodes. Prophylactic lateral neck dissection is controversial. In our case, we also performed left thyroid lobectomy and excision of left upper and lower parathyroid glands which were thought to be pathological. Parathyroid carcinoma had no invasion to thyroid lobe but micropapillary carcinoma 2 mm in diameter was detected in the excised thyroidectomy material.

In a study on the prognosis of the disease, 5 and 10-year survival rates were reported as 85.5% and 49.1%, respectively [16]. A study involving 224 patients reported a 10-year survival rate of 67.8% [1]. Although it is a rare cancer, many studies reported the most important prognostic factor as the success of en bloc resection performed in the initial surgical operation [14]. In the study by Koea and Shaw, in addition to R0 resection they performed, metastasis to lymph nodes and distant organs and non-functional status at the time of diagnosis were reported as poor prognostic factors [17]. Besides these factors, Harari et al. suggested that the number of recurrences and high calcium levels during recurrence were poor prognostic factors [17]. However, there are also studies suggesting that lymph node involvement was not a poor prognostic factor. Because regional lymph node metastasis are not common. But ipsilateral lymph node dissection must be performed if any lymph nodes are detected [18, 19].

## Conclusion

In our case with bifocal parathyroid carcinoma as a rare pathology, it should be considered that one of these focuses might have been originated from ectopic parathyroid tissue. Furthermore, our case was unique in that the disease was non-functional and the patient had concurrent micropapillary carcinoma detected on ipsilateral thyroidectomy specimen, which have not been reported before in the literature. It should be remembered that all of these variables may be present in the same patient, diagnosis and treatment algorithm should be arranged considering this situation.

## Competing interests

The authors declare no competing interest.
